# Apigenin suppresses the stem cell-like properties of triple-negative breast cancer cells by inhibiting YAP/TAZ activity

**DOI:** 10.1038/s41420-018-0124-8

**Published:** 2018-11-20

**Authors:** Ying-Wei Li, Jian Xu, Guo-Yuan Zhu, Zhu-Juan Huang, Yan Lu, Xian-Qian Li, Neng Wang, Feng-Xue Zhang

**Affiliations:** 1 Tropical Medicine Institute, Guangzhou University of Chinese Medicine, 510006 Guangzhou, P.R. China; 2 State Key Laboratory of Quality Research in Chinese Medicine, Macau Institute for Applied Research in Medicine and Health, Macau University of Science and Technology, Macau, P.R. China; 3 The Research Center for Integrative Medicine, Guangzhou University of Chinese Medicine, 510006 Guangzhou, P.R. China; 4 School of Basic Medicine Science, Guangzhou University of Chinese Medicine, 510006 Guangzhou, P.R. China; 5 Guangdong Provincial Academy of Chinese Medical Sciences, Guangzhou University of Chinese Medicine, 510006 Guangzhou, P.R. China

## Abstract

Triple-negative breast cancer (TNBC) remains a clinical challenge because of the absence of effective therapeutic targets. In TNBC, overexpression of YAP and TAZ correlates with bioactivities of cancer stem cells (CSCs), high histological grade, resistance to chemotherapy, and metastasis. Thus, YAP/TAZ may serve as potential therapeutic targets in TNBC. To identify YAP/TAZ inhibitors, in previous experiments, we screened a library of natural compounds by using YAP/TAZ luciferase reporter assay and identified apigenin as a potential inhibitor. In this study, we demonstrated that apigenin significantly suppressed the proliferation and migration of TNBC cells. Furthermore, we demonstrated that apigenin inhibited stemness features of TNBC cells in both in vitro and in vivo assays. Our mechanism study demonstrated that apigenin decreased YAP/TAZ activity and the expression of target genes, such as CTGF and CYR61, in TNBC cells. We also showed that apigenin disrupted the YAP/TAZ-TEADs protein–protein interaction and decreased expression of TAZ sensitized TNBC cells to apigenin treatment. Collectively, our studies suggest that apigenin is a promising therapeutic agent for the treatment of TNBC patients with high YAP/TAZ activity.

## Introduction

Breast cancer is the most frequently diagnosed cancer in women worldwide^1,2^. It is highly heterogeneous and clinically classified into different subtypes based on the status of receptors^3^. Triple-negative breast cancer (TNBC) is one of the most aggressive breast cancer subtypes and constitutes around 15–20% of breast cancer cases^4^. Because of the lack of therapeutic targets, patients diagnosed with this type of breast cancer have a poor prognosis, along with a high rate of recurrence after chemotherapy^5,6^. Hence, the development of effective treatments for TNBC is an important unmet medical need.

YAP and TAZ, two main downstream effectors of the Hippo pathway, are overexpressed or aberrantly activated in various malignancies^7,8^. Abnormal activation of YAP/TAZ is involved in several biological processes, such as epithelial-mesenchymal transition (EMT), tumor metastasis, and tumorigenesis^9–13^. The most recent studies also demonstrate that YAP/TAZ is required to sustain the self-renewal and tumor-initiation capacities of cancer stem cells (CSCs)^14,15^. Particularly in TNBC, high YAP/TAZ activity correlates with bioactivities of CSCs, high-grade histology, and metastasis^16–21^. Thus, identifying new potential therapeutic agents that inhibit YAP/TAZ activity is meaningful for the treatment of TNBC.

Natural products and their derivatives have been used as anticancer agents and present a potential source of new drugs to combat cancer^22–24^. To identify potential YAP/TAZ inhibitors, we previously performed a YAP/TAZ luciferase reporter activity-based screening of a library of natural products (Selleckchem, Houston, TX, USA), and found that apigenin decreased YAP/TAZ activity. In this study, we demonstrated that apigenin significantly suppressed the migration and CSCs properties of TNBC cells. We found that apigenin acted at least partially by inhibiting the YAP/TAZ-TEADs complex activity. These results confirmed that apigenin was a promising agent for the treatment of TNBC patients with high YAP/TAZ activity.

## Results

### Apigenin inhibited cell viability in TNBC cells

**Fig. 1:**
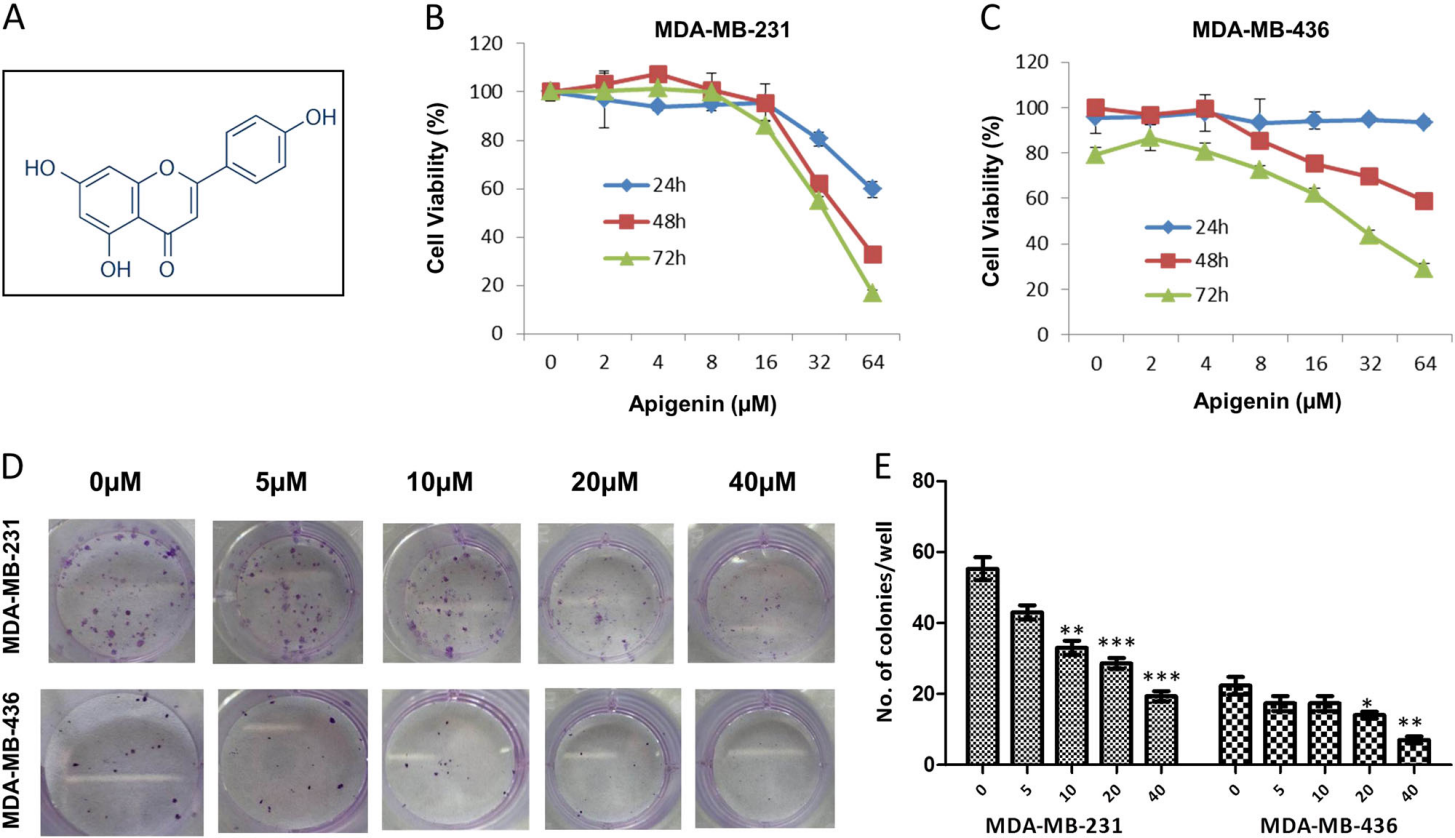
Apigenin inhibited proliferation and colony formation of TNBC cells. **a** Chemical structure of apigenin. **b**, **c** MDA-MB-231 and MDA-MB-436 cells were treated with apigenin (0–64 μM) for 24, 48, and 72 h, and cell viability was measured via the SRB assay. IC50 values were calculated by Prism 6 software. **d** Representative images of colony formation assay of the two cell lines. MDA-MB-231 and MDA-MB-436 cells were plated in 24-well plates and treated with indicated concentration of apigenin for 72 h, and then replaced for fresh medium. After 10 days, the plates were stained with 0.2% crystal violet and counted. **e** Summary of the number of colonies in MDA-MB-231 and MDA-MB-436 cells. The results shown are representative of at least three independent experiments. Data are shown as the mean ± S.D. (*P < 0.05 and **P < 0.01 compared with the control group)

We used the SRB protein assay to investigate the effect of apigenin on the proliferation of TNBC cells and found that apigenin inhibited cell proliferation in a dose- and time-dependent manner (Fig. 1b, c). The IC50 values of apigenin in MDA-MB-231 and MDA-MB-436 cells were approximately 33 and 30 μM, respectively, at 72 h. As shown in Supplementary Fig. 1, apigenin was relatively nontoxic to normal human breast cells. We also examined whether apigenin might affect the ability of TNBC cells to form colonies and found that apigenin indeed significantly reduced the rate of colony formation in both TNBC cell lines (Fig. 1d, e).

### Apigenin attenuated migration in TNBC cells

**Fig. 2:**
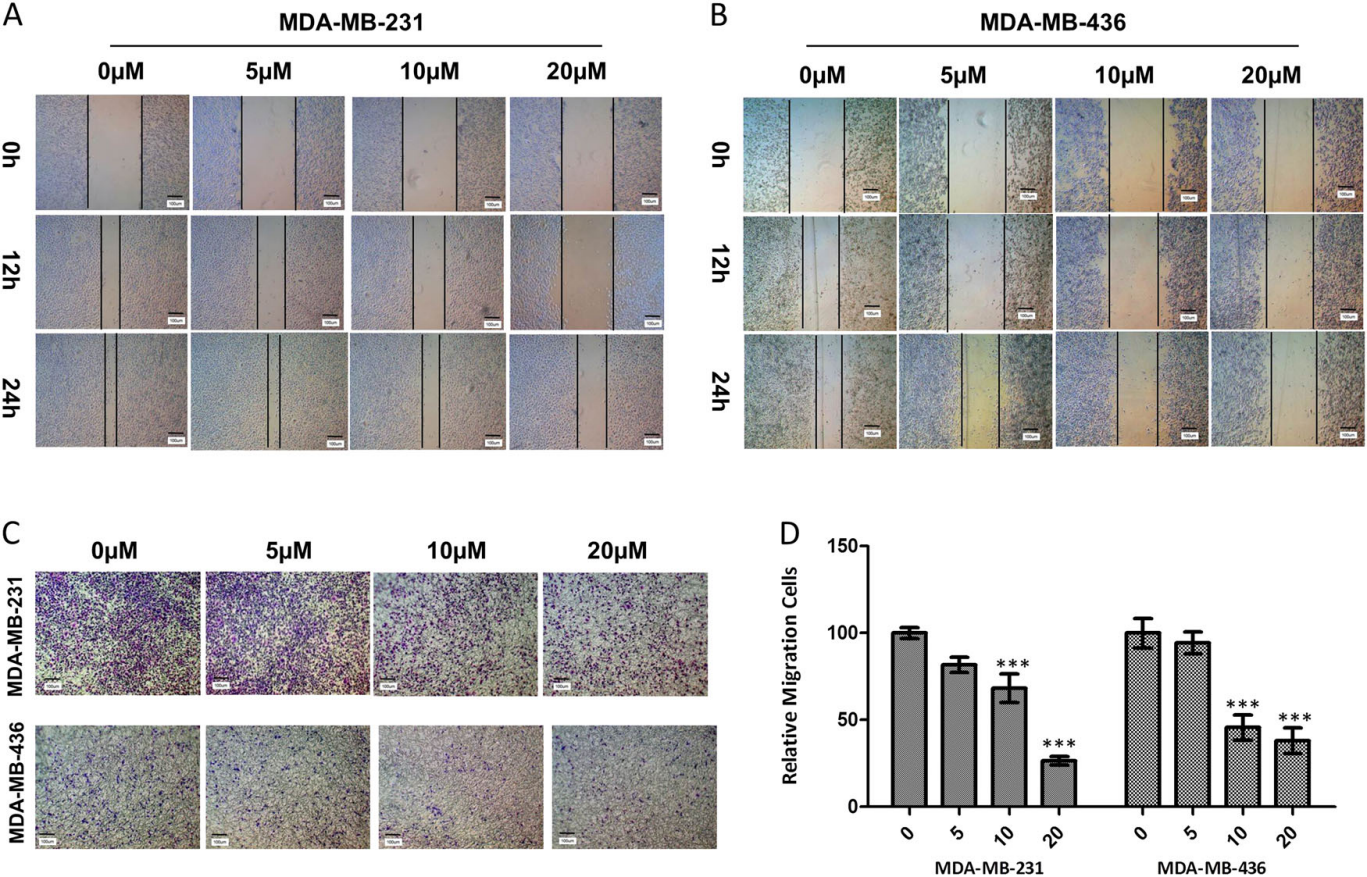
Apigenin attenuated migration of TNBC cells. **a**, **b** Representative images from the wound-healing assay of MDA-MB-231 and MDA-MB-436 cells are displayed for 12 and 24 h. **c** Representative images of the transwell migration assay are shown for 24 h after cells seeding. **d** Statistical analysis of relative migrating cell numbers. The results shown are representative of at least three independent experiments. Data are shown as the means ± S.D. (**P < 0.01 and ***P < 0.001 compared with the control group)

We used wound-healing assays to evaluate the effects of apigenin on the migration of TNBC cells. Our results demonstrated that apigenin reduced gap closure at concentrations of 10 and 20 µM (Fig. 2a, b). We also conducted transwell migration assays on TNBC cells to confirm the above finding. As shown in Fig. 2c, apigenin treatment decreased the number of migrated cells in a dose-dependent manner. Statistical analysis indicated that apigenin caused a significant decrease in the migration of MDA-MB-231 and MDA-MB-436 cells (Fig. 2d).

### Apigenin suppressed the stem cell-like properties and tumorigenic potential of TNBC cells

**Fig. 3:**
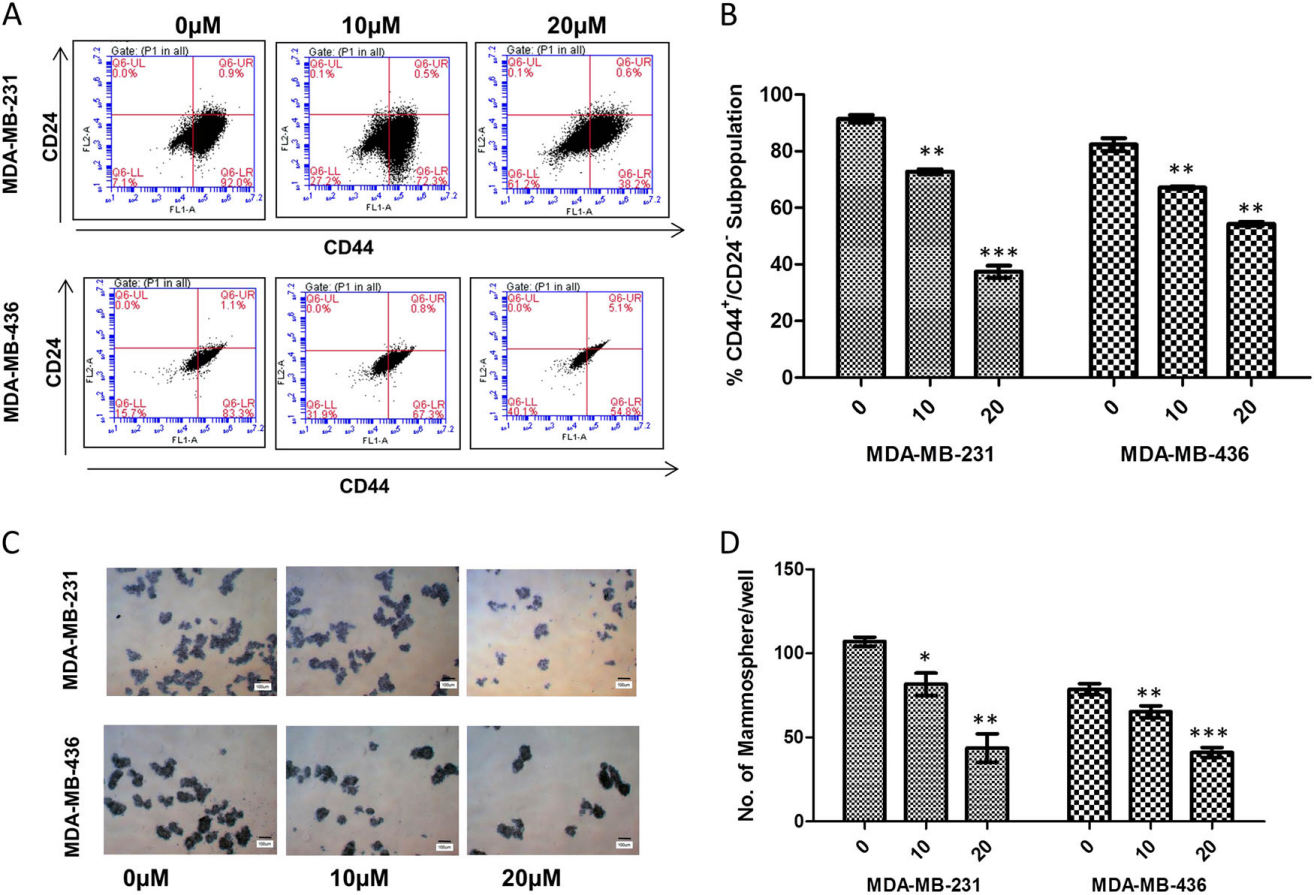
Apigenin inhibited breast cancer stem cell (CSC) properties. **a** Representative dot plots of CD44^+^/CD24^−^ subpopulation in MDA-MB-231 and MDA-MB-436 cells. Breast cancer cells were incubated with 10 or 20 μM apigenin for 72 h. CD44-FITC and CD24-PE antibodies were utilized to detect breast CSC population by using the BD Accuri C5. **b** Summary of the percentage of CD44^+^/CD24^−^ subpopulation in MDA-MB-231 and MDA-MB-436 cells with or without apigenin treatment. (**P < 0.01 and ***P < 0.001 compared with the control group). **c** Apigenin decreased mammosphere formation of MDA-MB-231 and MDA-MB-436 cells. MDA-MB-231 and MDA-MB-436 cells were treated with indicated concentrations of apigenin for 72 h, and mammosphere formation assay was performed by plating 10,000 cells into ultra-low attachment 6-well plates, and cultured for 10 days. **d** Quantification of mammosphere formation treated in the DMSO vehicle or apigenin (10 and 20 µM). Bars denote standard errors (*P < 0.05; **P < 0.01; ***P < 0.001)

We examined the anti-CSCs effects of apigenin over a range of concentrations that did not cause massive cell death (0–20 µM). We first investigated whether apigenin could decrease the percentage of the CD44^+^/CD24^−^ subpopulation in TNBC cells. As shown in Fig. 3a, b, apigenin treatment decreased the CD44^+^/CD24^−^ CSC subpopulations in MDA-MB-231 and MDA-MB-436 cells. Consistent with these findings, we observed that apigenin significantly decreased the number of mammospheres formed from both cell lines, indicating a reduction in self-renewal capability (Fig. 3c, d). These results indicated that apigenin decreased CSC-like traits on TNBC cells in vitro.

**Fig. 4:**
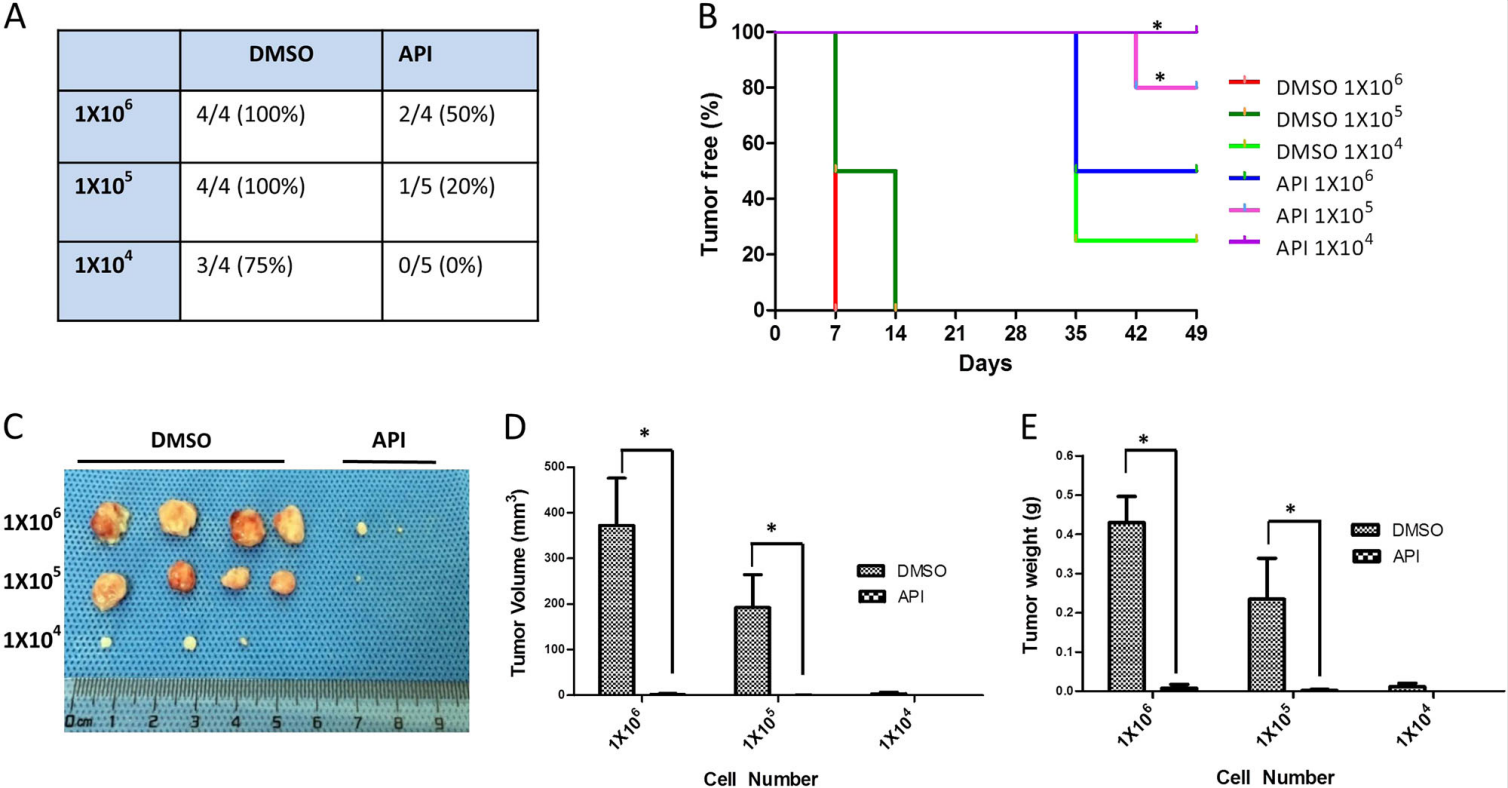
Apigenin inhibited CSCs in vivo according to the limited dilution assay. MDA-MB-231 cells were treated with DMSO vehicle or apigenin (20 µM) for 48 h. Cells were injected subcutaneously into the right front axilla of female BALB/c nude mice. Tumorigenesis was recorded after 7 days. **a** The incidence of tumors in nude mice is listed based on the different cell numbers that were seeded. **b** The tumor-free survival curves of the nude mice that were inoculated with different numbers of MDA-MB-231 cells with and without apigenin treatment are shown. The statistical significance was determined by log-rank test (*P < 0.05). **c** The tumors harvested at the end of the experiment. **d** The tumor volumes at the end of the experiment. Tumor size was measured by calipers, and tumor volumes were calculated according to the formula LW^2^/2. **e** Tumor weights at the end of the experiment. The statistical significance was determined by Student’s *t*-test. The difference between apigenin and DMSO treatment is significant (***P < 0.001)

We also examined the effects of apigenin on the tumor-initiating properties of TNBC cells using an in vivo limited dilution assay. As shown in Fig. 4a, 1 × 10^6^ and 1 × 10^5^ MDA-MB-231 cells formed tumor xenografts with 100% efficiency in the control group, but the tumor formation efficiency of the apigenin-treated group decreased to 50% and 20%, respectively. When cells were seeded at a density of 1 × 10^4^ cells per site, the tumor formation efficiency in the apigenin-treated group decreased to 0%, whereas the control group retained 75% of its efficiency. Additionally, the onset of tumor growth in the apigenin-treated group was delayed compared with the control, DMSO-treated group (Fig. 4b). Apigenin treatment also significantly decreased tumor volumes and weights (Fig. 4c–e).

### Apigenin reversed the malignant phenotype of TNBC cells by inhibiting YAP/TAZ transcription activities

**Fig. 5:**
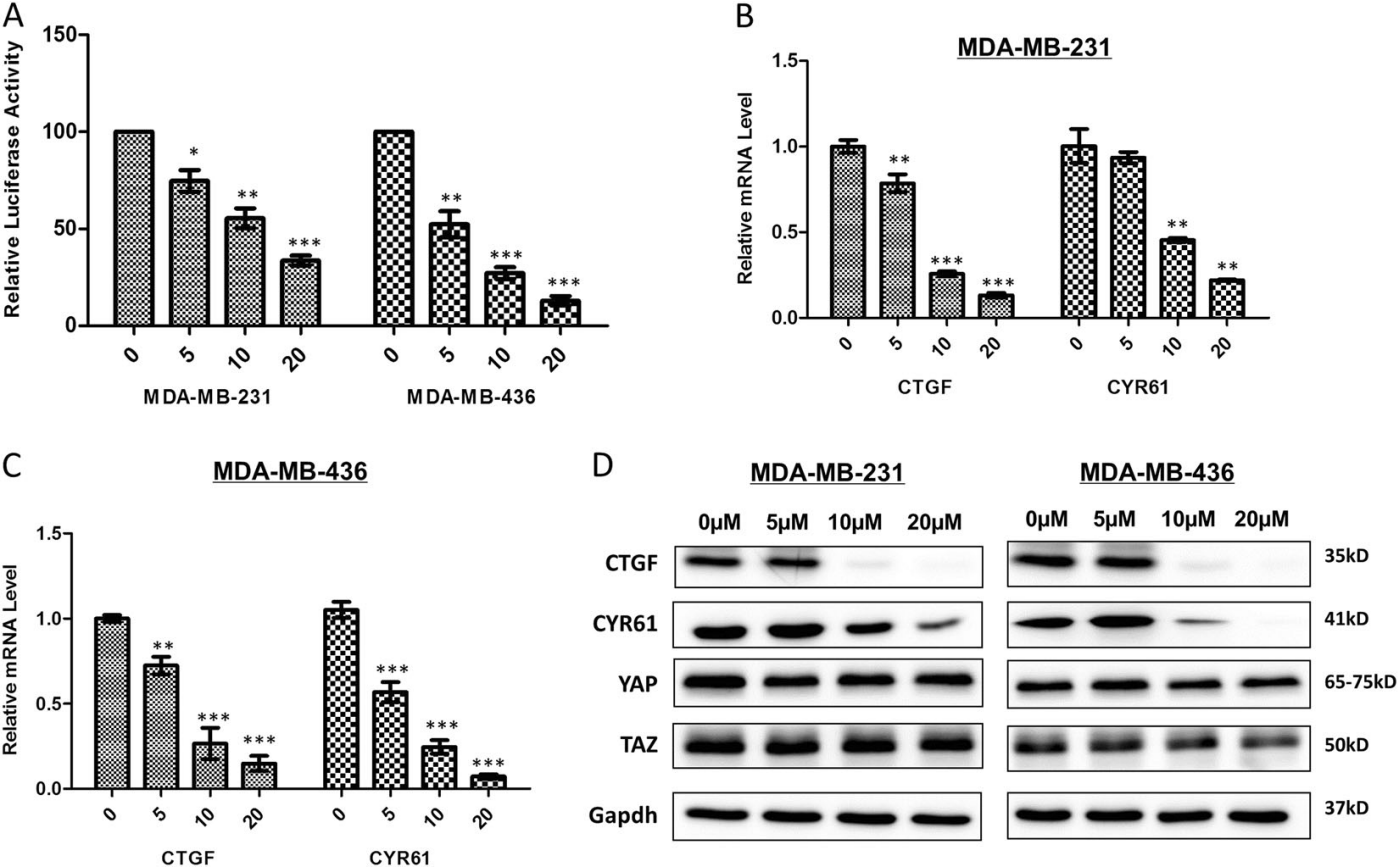
Apigenin decreased YAP/TAZ transcriptional activity in TNBC cells. **a** Apigenin reduced YAP/TAZ luciferase reporter activity in MDA-MB-231 and MDA-MB-436 cells. **b**, **c** Apigenin reduced mRNA level of CTGF and CYR61 in the two cell lines. Each experiment was performed in triplicate. The results shown are representative of at least three independent experiments. Data are shown as the means ± S.D. (**P < 0.01 and ***P < 0.001 compared with the control group). **d** CTGF and CYR61 expression in apigenin-treated TNBC cells were revealed by immunoblot. Gapdh was used as loading control. The results shown are representative of at least three independent experiments

To explore whether apigenin inhibited YAP/TAZ transcriptional activity in TNBC cells, we used the luciferase reporter assay. Our results demonstrated that apigenin significantly decreased YAP/TAZ activity in TNBC cells (Fig. 5a). To further confirm that apigenin inhibited YAP/TAZ transcriptional activity, we evaluated the expression of the YAP/TAZ target genes, CTGF and CYR61, and found that apigenin significantly decreased the mRNA and protein levels of CTGF and CYR61 in a dose-dependent manner (Fig. 5b–d). The protein levels of YAP and TAZ, however, were not affected by apigenin treatment.

### Apigenin disrupted YAP/TAZ–TEAD interaction in TNBC cells

**Fig. 6:**
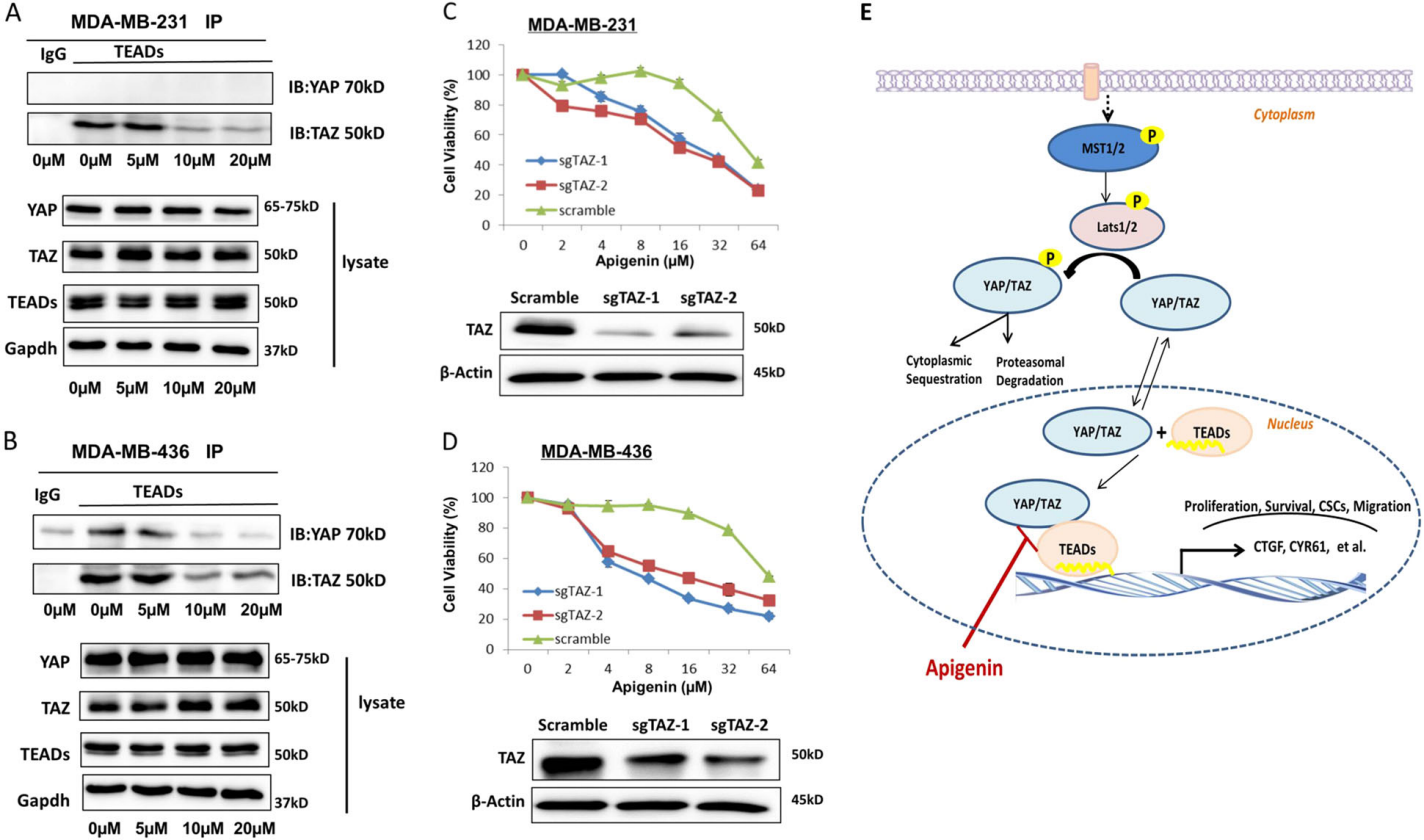
Apigenin disrupted YAP/TAZ and TEAD interaction in TNBC cells. **a**, **b** MDA-MB-231 and MDA-MB-436 cells were treated with DMSO vehicle or apigenin (5, 10, and 20 µM) for 72 h. The cells were harvested, and the effect of apigenin on the YAP/TAZ and TEADs interaction was analyzed by immunoprecipitation assay. **c**, **d** TAZ knockout sensitized TNBC cells to apigenin treatment. Both Scramble and sgTAZ cells were treated with apigenin (0–64 μM) for 48 h, and cell viability was measured via the SRB assay. **e** Schematic diagram of the mechanism of apigenin-mediated antitumor activity by inhibiting YAP/TAZ activity in TNBC cells. Apigenin inhibited YAP/TAZ activity and decreased the expression of downstream genes by targeting YAP/TAZ-TEAD protein–protein interaction

We previously verified that the TAZ–TEADs interaction was indispensable for TAZ in maintaining CSCs traits of breast cancer cells^25^. Therefore, we hypothesized that apigenin could act by disrupting the interaction of TAZ and TEADs in TNBC cells, and we performed co-immunoprecipitation assays to explore the interaction between TAZ and TEADs. Apigenin disrupted the interaction of TAZ and TEADs in MDA-MB-231 and MDA-MB-436 cells (Fig. 6a, b), as well as the interaction of YAP and TEADs in MDA-MB-436 cells. To further explore whether TAZ played a major role in the anticancer activities of apigenin, we decreased TAZ expression in TNBC cells by CRISPR/Cas9-mediated genome editing. As expected, knockdown of TAZ sensitized TNBC cells to apigenin treatment, with a 50–70% decrease in IC50 (Fig. 6c, d). These results suggested that the reversion of the malignant phenotype of TNBC cells by apigenin was, at least in part, due to inhibition of the YAP/TAZ-TEADs complex activity.

## Discussion

The theory of CSCs proposed that a small population of cancer cells had features similar to normal stem cells and that these cells played important roles in tumor initiation and maintenance^26^. In recent studies, CSCs have been isolated and identified in several solid tumors, including breast cancer^27^. In TNBC, breast CSCs were highly enriched and related to chemotherapy resistance, tumor relapse, and metastasis^28–30^. Therefore, the development of therapy targeting CSCs might benefit patients with TNBC.

Apigenin is a widely distributed flavonoid in vegetables and fruits. Recent studies have reported that apigenin has anticancer activity^31–33^, but the effects and underlying mechanisms of apigenin on TNBC have remained largely unexplored. In our drug-screening study, we found that apigenin decreased YAP/TAZ luciferase reporter activity. Therefore, we hypothesized that apigenin inhibited the migration and properties of CSCs in TNBC cells by regulating the Hippo-YAP/TAZ signaling pathway.

The population of CD44^+^/CD24^−^ cells was recognized as CSCs in breast cancer^34^. In the current study, we used MDA-MB-231 and MDA-MB-436 cells to investigate the functions and mechanisms of apigenin on TNBC cells, because both cell lines overexpressed TAZ and possessed a high number of CD44^+^/CD24^−^ cells. Our results showed that apigenin significantly suppressed proliferation and migration of TNBC cells. We further demonstrated that apigenin robustly inhibited features of stemness in TNBC cells, evidenced by a decrease in the CD44^+^/CD24^−^ CSC subpopulation and mammosphere formation. Also, limiting dilution analysis of tumorigenesis confirmed that apigenin inhibited the tumor-initiating properties of TNBC in vivo.

Next, we investigated the molecular mechanisms underlying the anticancer effects of apigenin. We verified that apigenin inhibited YAP/TAZ transcriptional activity in TNBC cells, as assayed by luciferase reporter assays. Consistently, apigenin significantly decreased the expression of CTGF and CYR61, two YAP/TAZ-regulated genes, at both the mRNA and protein level.

YAP and TAZ mainly interact with TEADs to induce the expression of downstream genes that are involved in cell proliferation and migration^35–37^. Therefore, the YAP/TAZ-TEADs complex may serve as a potential therapeutic target. In the current study, we investigated the effect of apigenin on the interaction of YAP/TAZ and TEADs. Our results demonstrated that apigenin disrupted the YAP/TAZ-TEADs protein–protein interaction in MDA-MB-436 cells. In MDA-MB-231 cells, we found that apigenin disrupted the TAZ–TEADs interaction. We did not find evidence for interaction between YAP and TEADs in MDA-MB-231 cells. We assume that TAZ, but not YAP, plays a key role in the downstream effects of the Hippo pathway in MDA-MB-231 cells. Furthermore, CRISPR/Cas9-mediated knockout of TAZ sensitized TNBC cells to apigenin treatment.

In conclusion, we demonstrated for the first time that apigenin inhibited YAP/TAZ activity in TNBC cells. The effects of apigenin on TNBC cells are mediated, at least in part, by disrupting the YAP/TAZ-TEADs protein–protein interaction, which is depicted in the model shown in Fig. 6e. These results suggested that apigenin might offer a novel therapeutic option for TNBC patients with high YAP/TAZ activity.

## Materials and methods

### Antibodies and reagents

YAP, TAZ, TEADs, GAPDH, β-Actin, CTGF, CYR61, rabbit mAb IgG control and anti-rabbit IgG (Light-Chain Specific) were purchased from Cell Signaling (Danvers, MA, United States); anti-mouse IgG and anti-rabbit IgG were purchased from Bio-Rad (Bio-Rad, Hercules, CA, United States); CD24-PE and CD44-FITC antibodies from BD Biosciences (Franklin Lakes, NJ, USA); EGF and b-FGF was purchased from PeproTech, Inc. (Rocky Hill, NJ, USA), B27 and puromycin was from Thermo Fisher Scientific, Inc. (Waltham, MA, USA). BMStbl3 competent cells were from Biomed (Beijing, China); insulin, cholera toxin, hydrocortisone, ampicillin sodium salt, sulforhodamine B (SRB), LB broth, and LB broth with agar were purchased from MilliporeSigma (Burlington, MA, USA). 8xGTIIC-luciferase was a gift from Stefano Piccolo (Addgene plasmid # 34615). Apigenin (S2262, purity >99%) was obtained from Selleckchem (Houston, USA) and dissolved in sterile-filtered dimethyl sulfoxide (DMSO; MP Biomedicals, Santa Ana, CA, USA). Final DMSO concentration was 0.1% in Apigenin- and vehicle-treated cells. All consumables and regular reagents for the experiments were purchased from VWR Life Science (Radnor, PA, USA).

### Plasmid construction

We constructed the single guide RNAs (sgRNAs) targeting exon 1 of the human *WWTR1* gene (TAZ, NM_001168278) as previously described^38^. The target sequences for the TAZ guide were 5′-CGCGAGTGCGAGCCCGAATC-3′ and 5′-GCAAGTGATCCACGTCACGC-3′; the scrambled sequence was 5′-AACAGTCGCGTTTGCGACTGG-3′. The constructs were verified by sequencing. The extraction of plasmids was performed using EndoFree Mini Plasmid Kit according to the manufacturers’ protocol (TIANGEN, Beijing, China).

### Cell culture and transfection

MDA-MB-231 cell lines were purchased from American Type Culture Collection (Manassas, VA, USA). MCF-10A and MDA-MB-436 cell lines were obtained from Cell Bank of Chinese Academy of Sciences. MDA-MB-231 and MDA-MB-436 cells were cultured in Dulbecco’s modified Eagle medium (DMEM, GIBCO) containing 10% fetal bovine serum (FBS). MCF-10A cells were cultured as previously described^39^. Cells were maintained in a humidified atmosphere of 95% air and 5% CO2 at 37 °C. For knockout experiments, MDA-MB-231 and MDA-MB-436 were transfected with 2.5 μg of LentiCRISPR-v2-TAZ or 2.5 μg of LentiCRISPRv2-Scramble (control). Transfection was performed using Lipofectamine 3000 DNA Transfection Reagent (Thermo#L3000-015) following the manufacturer’s protocol. TAZ-edited cells and control cells were selected using 3 μg/ml puromycin (Gibco#A11138-03) for 48 h. The knockout of TAZ was validated by western blot.

### Cell proliferation and colony formation assay

SRB protein assay was used in this study to analyze cell proliferation^40^. Briefly, TNBC cells were plated in 96-well plates (6 × 10^3^ cells per well) and grown overnight. Apigenin (0–64 μM) was added to the indicated wells and plates were incubated for 24, 48, or 72 h. The IC50 value of apigenin in TNBC cells was calculated using GraphPad Software. For colony formation, MDA-MB-231 and MDA-MB-436 cells (1000 cells per well) were seeded on 24-well plates. The experimental groups were treated with the indicated concentrations of apigenin for 72 h and then replaced for fresh medium. The plates were finally stained with 0.2% crystal violet (Amresco#0528) and counted.

### Mammosphere formation assay

TNBC cells were treated with different concentrations of apigenin for 72 h and mammosphere formation assay was conducted as previously decribed^25^. In brief, apigenin-treated and DMSO-treated cells were plated into ultra-low attachment plates at the density of 10,000 cells per well (Corning, NY, USA). On day 10, images were taken at 4x magnification and counted.

### Flow cytometry analysis

MDA-MB-231 and MDA-MB-436 cells were treated with apigenin for 72 h. Cells were resuspended in phosphate-buffered saline (PBS) containing 0.5% FBS and stained with anti-CD24-PE (BD#555428) and anti-CD44-FITC (BD#555478) for 30 min at 4 °C in the dark. Cells were washed with PBS and then analyzed by flow cytometry.

### Wound-healing and transwell migration assays

TNBC cells were seeded onto 6-well plates to reach 90% confluence. A sterile P200 pipette tip was used to create a scratch in the middle of each well. Cell debris was removed, and the remaining cells were maintained in the absence or presence of apigenin for 24 h. Migrated cells were photographed at 0, 12, or 24 h by using a Zeiss inverted microscope (Zeiss GmbH, Germany; magnification, 4 × ). For transwell migration assay, TNBC cells were pretreated with the indicated concentrations of apigenin for 24 h. Cells were resuspended in serum-free medium, plated into transwell chambers (8-µm pore size; Corning), and cultured for 24 h. Migrated cells were stained with crystal violet and counted under the microscope.

### Luciferase reporter assay

Luciferase reporter assay was performed using previously described methods^41^. Briefly, MDA-MB-231 or MDA-MB-436 cells were plated in 96-well plates and grown overnight. Cells were co-transfected with 0.2 μg of 8xGTIIC-luciferase reporter plasmid and 10 ng of pRL-CMV plasmid using Lipofectamine 3000 reagent. Indicated concentrations of apigenin (5, 10, and 20 µM) were added to the cells at 24 h post-transfection. Luciferase activity was detected with the DLR™ assay system (Promega#E1910), according to the manufacturers’ protocol.

### Real-time PCR

TRIzol reagent (Thermo, USA) was applied for total RNA extraction. The RNA was reverse transcribed using the cDNA Synthesis Kit (Thermo#K1622) following the manufacturer’s instructions. We performed real-time polymerase chain reaction (PCR) analysis using the Power SYBR Green PCR Master Mix Power (Roche, Mannheim, Germany) on a Bio-Rad CFX96 detection system. The mRNA levels of target genes were standardized to the level of β-Actin. Primer sequences (in the 5′–3′direction) are as follows: CTGF-F: GCAGAGCCGCCTGTGCATGG; CTGF-R: GGTATGTCTTCATGCTGG; CYR61-F: CACACCAAGGGGCTGGAATG; CYR61-R: CCCGTTTTGGTAGATTCTGG; β-Actin-F: GGTGAAGGTCGGAGTCAACGG; β-Actin-R: GAGGTCAATGAAGGGGTCATTG.

### Western blotting and co-immunoprecipitation analysis

Cell lysates were collected with RIPA buffer containing protease inhibitors (Thermo Fisher Scientific) and cleared by centrifugation. BCA Assay (Thermo#23227) was applied for the determination of the protein concentration. Western blotting was performed according to the standard protocol. Protein bands were finally detected using the ECL detection reagents (Thermo Scientific#34580). For co-immunoprecipitation analysis, 500 μg proteins were incubated overnight with specific primary antibodies TEADs (CST#13295) or rabbit mAb IgG control (CST#3900) at 4 °C. Immune complexes were precipitated with Protein A/G PLUS-Agarose (Santa Cruz #sc-2003) and boiled with Laemmli sample buffer. Elutes were analyzed by Western blotting assay. The following primary antibodies were used in this study: YAP (CST#4912), TAZ (CST#70148), TEADs (CST#13295), GAPDH (CST#2118), β-Actin (CST#3700), CTGF (CST#86641), CYR61 (CST#14479), and YAP/TAZ (CST#8418).

### Tumorigenic evaluation assays

MDA-MB-231 cells were pretreated with DMSO or 20 µM apigenin for 48 h. Living cells were quantified by using trypan blue exclusion assay. Cells were suspended in PBS containing 50% Matrigel (BD#354230). A series dilution of apigenin-treated and DMSO-treated cells (10^6^, 10^5^, 10^4^) was injected into the right flank of female BALB/c nude mice (5–6-weeks-old; Charles River, Beijing, China). Tumor formation efficiency and tumor sizes were recorded once a week. Tumor volumes were calculated according to the formula of Length × Width^2^/2. The care and use of animals were approved by the Animal Care and Use Committee of Guangzhou University of Chinese Medicine (Guangzhou, China).

### Statistical analysis of data

GraphPad Prism 5 was used for statistical analyses. Student’s *t*-test was used for comparison between groups. Data are expressed as mean ± SD. Statistical significance was set at *P* < 0.05.

## Electronic supplementary material

Supplementary Figure 1

Supplementary Figure 2

Supplementary figure legends
