# Supplementary figures and images for: Apigenin suppresses the stem cell-like properties of triple-negative breast cancer cells by inhibiting YAP/TAZ activity
Source: Cell Death Discov. 2018 Nov 20;4:105. doi: 10.1038/s41420-018-0124-8 (PMC6244166; doi:10.1038/s41420-018-0124-8)

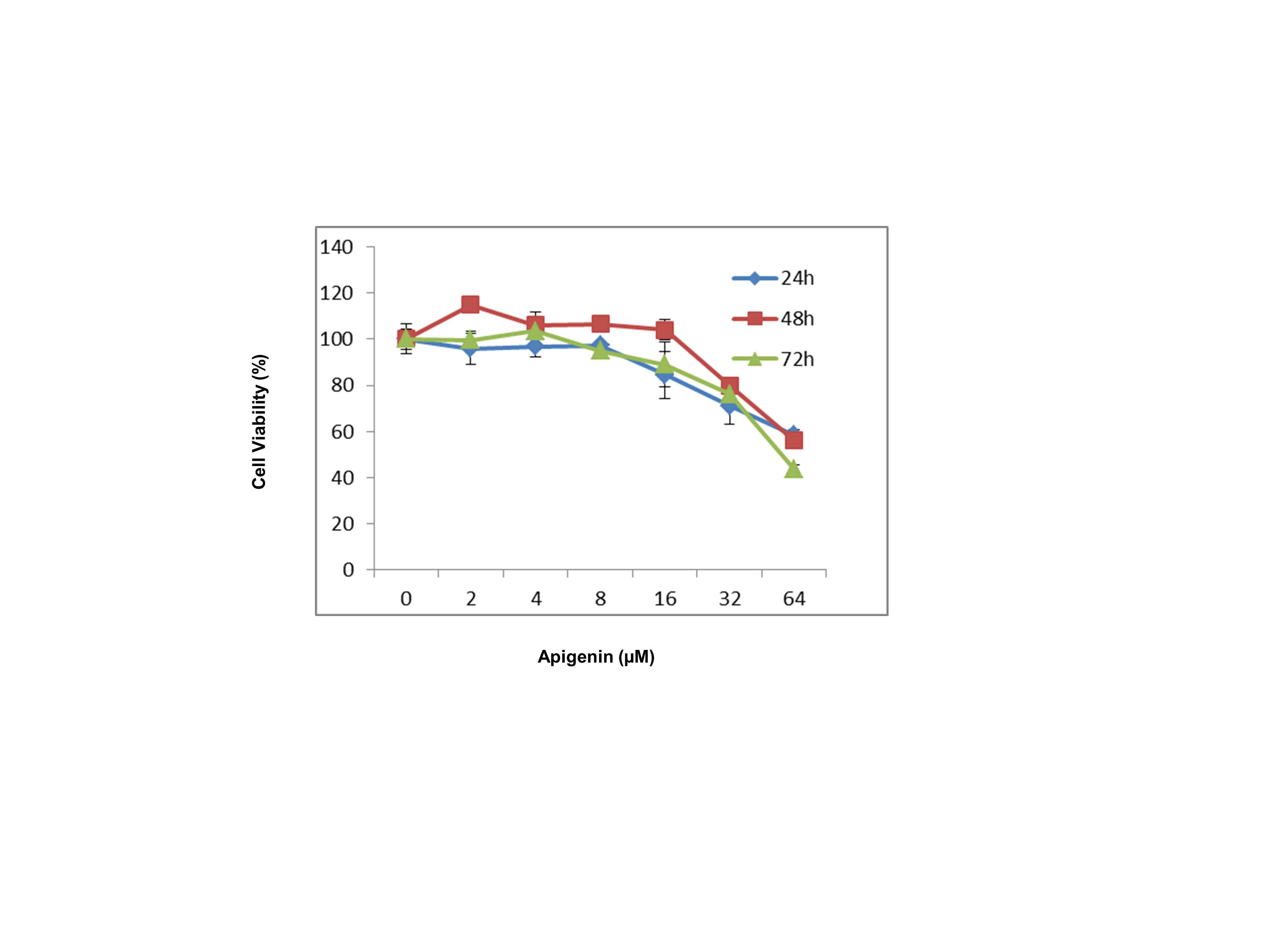

Supplement: Supplementary file 1 — Supplementary Figure 1 [file 41420_2018_124_MOESM1_ESM.jpg]

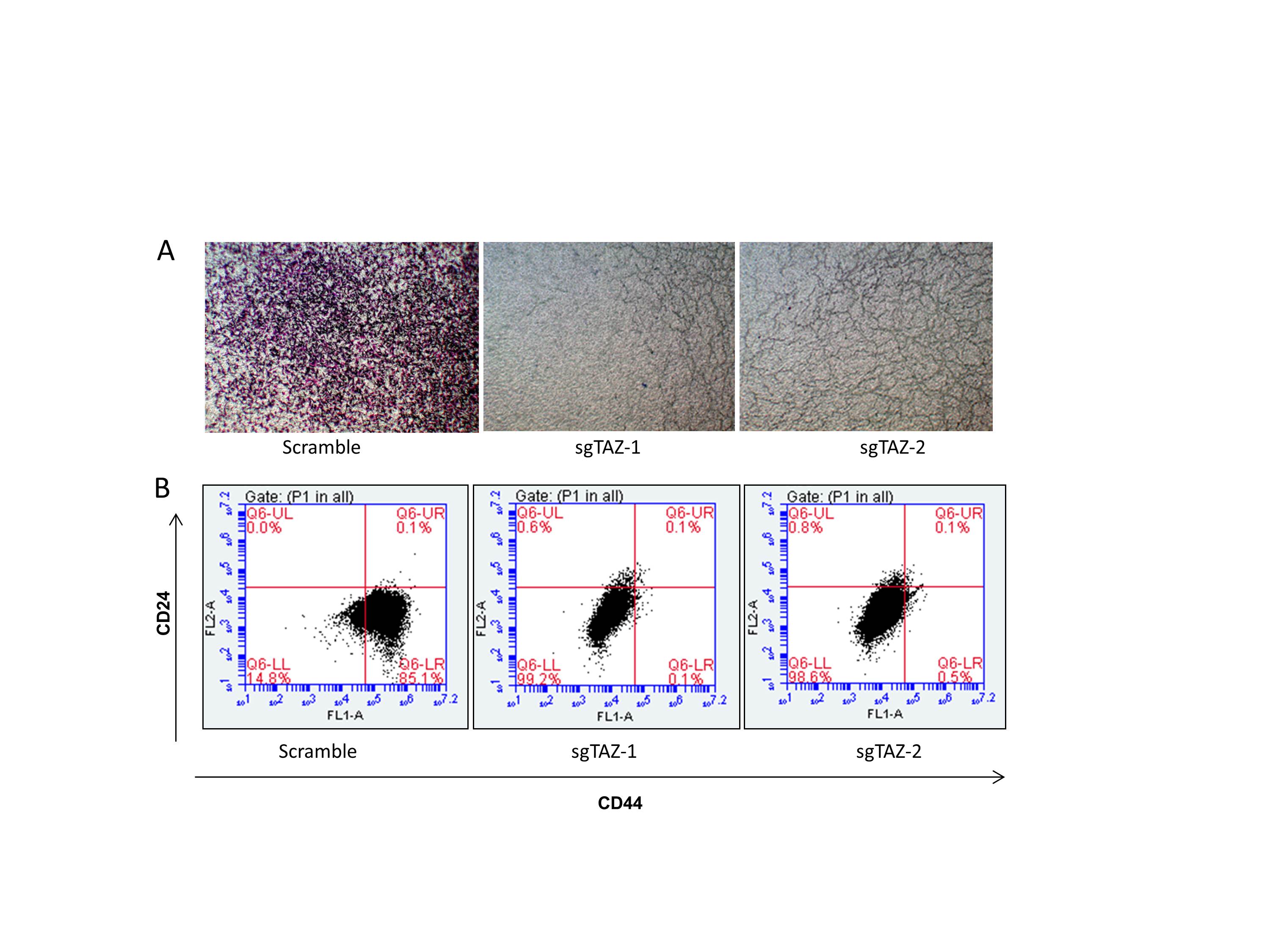

Supplement: Supplementary file 2 — Supplementary Figure 2 [file 41420_2018_124_MOESM2_ESM.jpg]
