# Supplementary material for: Apigenin suppresses the stem cell-like properties of triple-negative breast cancer cells by inhibiting YAP/TAZ activity
Source: Cell Death Discov. 2018 Nov 20;4:105. doi: 10.1038/s41420-018-0124-8 (PMC6244166; doi:10.1038/s41420-018-0124-8)
Supplement: Supplementary file 3 — Supplementary figure legends [file 41420_2018_124_MOESM3_ESM.doc]

**Supplementary Figure 1: Effects of apigenin on the proliferation of MCF10A cells.** MCF10A cells were treated with apigenin (0–64 μM) for 24, 48 and 72 h, and cell viability was measured via the SRB assay.

**Supplementary Figure 2: TAZ knockdown decreased cell migration and CSCs subpopulation in MDA-MB-231 cells. a** Representative images of the transwell migration assay are shown for 24h after cells seeding. **b** Representative dot plots of CD44^+^/CD24^−^ subpopulation in MDA-MB-231-Scramble and MDA-MB-231-sgTAZ cells. CD44-FITC and CD24-PE antibodies were utilized to detect breast CSC population by using BD Accuri C5.
